# Current and emerging brain applications of MR-guided focused ultrasound

**DOI:** 10.1186/s40349-017-0105-z

**Published:** 2017-10-05

**Authors:** Ying Meng, Suganth Suppiah, Karim Mithani, Benjamin Solomon, Michael L. Schwartz, Nir Lipsman

**Affiliations:** 1 Division of Neurosurgery, Department of Surgery, Sunnybrook Health Sciences Centre, University of Toronto, 2075 Bayview Avenue, Toronto, ON Canada; 2 Physical Sciences Platform, Sunnybrook Research Institute, Toronto, ON Canada

**Keywords:** Focused ultrasound, MRI guided focused ultrasound, MRgFUS, Neuroablation, Blood-brain barrier disruption, Neurologic disorders

## Abstract

MRI guided focused ultrasound is an emerging technique that uses acoustic energy to noninvasively treat intracranial disorders. At high frequencies, it can be used to raise tissue temperatures and ablate discrete brain targets with sub-millimeter accuracy. This application is currently under investigation for a broad range of clinical applications, including brain tumors, movement disorders, and psychiatric conditions. At low frequencies MRI guided focused ultrasound can be used to modulate neuronal activity and in conjunction with injected microbubbles, can open the blood-brain barrier to enhance the delivery of therapeutic compounds. The last decade has seen dramatic advances in the science of MRI guided focused ultrasound, helping elucidate both its mechanisms and potential in pre-clinical models, and its translational promise across myriad clinical applications. This review provides an update of current and emerging MRI guided focused ultrasound applications for intracranial disorders and describes future directions and challenges for the field.

## Background

The ability to focus acoustic energy through the intact skull on discrete brain targets has been a goal of ultrasound research for decades. Early efforts at therapeutic ultrasound in the brain, pioneered by the Fry brothers in the early 1950’s, though successful, required removal of the overlying skull due to the bone’s absorption and reflection of a large portion of ultrasound energy [1]. In the late 1990s, completely noninvasive treatment was realized with the development of a helmet device lined with multiple independent transducers, and the coupling of FUS to real-time magnetic resonance (MR) image guidance [2]. The MR guided FUS (MRgFUS) design allowed computer calculated and controlled steering of each element to achieve accurate targeting. The device is further coupled with thermography to allow real-time feedback on the effect of sonications. In one commercially available design, a spherical helmet device harnesses acoustic energy from over 1000 individual transducer elements operating between 650 and 720 kHz. Currently, the patient’s head must be stabilized in a stereotactic frame and their hair must be shaved to allow good coupling between the transducers and scalp. Other devices may use fewer transducers, as well as the possibility to implant the system in the skull for ongoing ultrasound application [3].

**Fig. 1:**
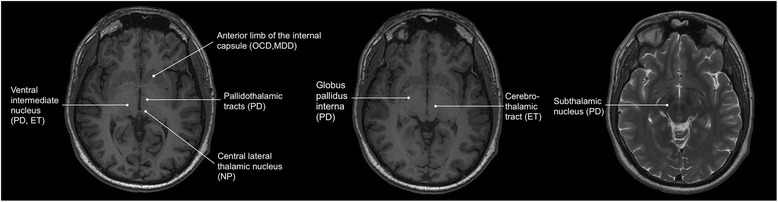
Schematic of intracranial regions targeted by MRgFUS in published human studies on an axial T1 and T2 weighted 3 Tesla MR images. OCD = obsessive compulsive disorder; MDD = major depressive disorder; PD = Parkinson’s disease; ET = essential tremor; NP = neuropathic pain

Over the last decade the potential applications of MRgFUS in neurosurgery have significantly expanded. MRgFUS was recently approved by the US Food and Drug Administration (FDA) to perform thalamotomy for patients with medically refractory essential tremor (ET), and is currently under investigation for treatment of brain tumors, as well as treatment resistant psychiatric conditions (Fig. 1) [4]. Moreover, a recent cost-effective analysis suggested that MRgFUS may be cost-effective compared to other neuromodulation modalities, such as deep brain stimulation or stereotactic radiosurgery for the treatment of ET [5]. Currently, there are more than 25 focused ultrasound clinical trials publically registered for patients with neurological disorders ranging from neuro-oncology, Parkinson’s disease, Alzheimer’s disease, obsessive-compulsive disorder, and epilepsy (Table 1). This review provides an update of MRgFUS research in the preclinical and clinical realm within the last decade, and describes the challenges ahead and future directions for the field.

**Table 1:** List of clinical trials investigating neurological applications of MRgFUS by disorder

Indication	Stage	Description
ET	Phase II	NCT02289560 – multi-centered, single arm study to look at efficacy for ET NCT01827904 - prospective, randomized, double blind, crossover, multi-centered, two arm study to test efficacy and further demonstrate safety
PD	Phase I	NCT02347254, NCT01772693, NCT02263885, NCT02246374, NCT02252380 - phase I trial to study unilateral Vim nucleus/subthalamic nucleus/globus pallidus sonication for PD
Brain tumor – ablation	Phase I	NCT00147056, NCT02343991, NCT01473485 – phase I trial for tumor ablation, ongoing NCT01698437 – phase I trial for tumor ablation, completed, University Children’s Hospital in Zurich
Brain tumor – BBB disruption	Phase I	NCT02343991 – phase I trial to study BBB opening for delivery of doxorubicin
Depression/anxiety	Phase I	NCT02348411 – phase I trial to study safety and initial effectiveness of MRgFUS bilateral anterior capsulotomy in medication-refractory MDD NCT02685488 – phase I trial of right frontotemporal area sonication for mild to moderate depression
OCD	Phase I	NCT01986296 - phase I trial of anterior cingulate cortex, anterior limb of internal capsule, ventral striatum or subgenual cingulate cortex sonication NCT03156335 - phase I trial to evaluate the safety and efficacy of MRgFUS for patients with treatment-refractory OCD
Pain syndromes	Phase I	NCT01699477 - phase I trial of thalamic sonication for neuropathic pain, pediatric, University Children’s Hospital in Zurich Central lateral thalamic nucleus sonication (Neurosurgery Focus 2012) NCT03111277 - phase I trial for MRgFUS thalamotomy of central lateral thalamic nucleus
Epilepsy – ablation	Phase I	NCT02804230 – phase I trial, ablation of subcortical focal epileptic targets
Epilepsy - neuromodulation	Phase I	NCT02151175 – phase I trial, stimulation or suppression of neuronal activity in temporal lobe
AD	Phase I	NCT02986932 – phase I trial of ultrasound mediated BBB opening for AD
Thrombolysis/ICH	Preclinical	Swine and human cadaveric models demonstrated feasibility of ICH liquefaction. Rabbit carotid occlusion model demonstrated feasibility of this model for vascular recanalization
CSF diversion	Preclinical	Proof-of principle study of MRgFUS third ventriculostomy
Neuromodulation	Phase I	Vagus nerve modulation can stimulate or dampen neuronal activity

*ET* essential tremor, *PD* Parkinson’s disease, *BBB* blood-brain barrier, *MDD* major depressive disorder, *OCD* obsessive compulsive disorder, *AD* Alzheimer’s disease, *CSF* cerebrospinal fluid, *FUS* focused ultrasound, *ICH* intracerebral hemorrhage, *NCT* national clinical trial (number)

### Movement disorders

The application of MRgFUS to movement disorders has been among the most promising to date. Targets for movement disorder symptoms, such as tremor, are near the geographic center of the skull in key motor relay nuclei, making these amenable to heating with minimal heating of the overlying skull. In addition, the ability to monitor patient symptoms in real-time, and evaluate the impact of a lesion on tremor, provides a powerful behavioral assay on which to base treatment decisions. To date, essential tremor (ET) and Parkinson’s Disease (PD) have been the primary applications, owing in large part to the large experience with these conditions in the ablative neurosurgical literature.

#### Essential tremor

Approximately 4% of people over 40 years of age are affected by essential tremor, which is marked by postural and intention tremors of about 4–7 Hz [6]. In some patients, these tremors can be severe enough to affect daily activities such as eating, drinking, and writing. 15–25% of patients are forced to retire prematurely as a result of ET, and 60% of patients do not apply for jobs or promotions due to uncontrollable shaking [7]. First-line therapy for ET is medical, typically with propranolol and primidone. However, one third of the patients will find the side effects of these medications intolerable or have refractory symptoms. Alternative treatments for ET includes surgical targeting of the ventral intermediate nucleus (Vim) of the thalamus. The Vim is part a circuit that connects the cerebellum with cortical motor pathways important for tremor. While deep brain stimulation electrodes are implanted bilaterally [8], the target contralateral to the more disabled side is treated in radiofrequency (RF) ablation to minimize severe neurological complications [9]. These two modalities are the most well-established interventions in the treatment of ET. While they are effective, both involve cranial access, which carries a risk of infection and hemorrhage. The rates of infection, seizures, and intracranial hemorrhage after DBS, which may be taken as correlates to brain penetration, are 1.7%, 1.5% and 3.9% respectively [10]. Device-related issues such as hardware breakage, mal-placement or migration, and battery depletion make life-long maintenance necessary for DBS. Gamma knife radiosurgery is another ablative technique. Despite being noninvasive, radiosurgery is less favorable because the results are variable and delayed often requiring weeks to months to become noticeable.

Two pilot studies of focused ultrasound Vim ablation were published in 2013 demonstrating promising clinical results after MRgFUS thalamotomy [11, 12]. In one study of fifteen patients, the Clinical Rating Scale for Tremor (CRST) score improved by 74% at 12 months (*p* = 0.001) and disability score by 85% at 12 months (p = 0.001) [11]. A subsequent multicenter randomized sham control trial in 76 patients with ET showed that MRgFUS treatment was superior to placebo with 47% improvement in hand-tremor score at 3 months and 62% reduction in disability score [4]. More recently, Zaaroor et al. reported their experience with MRgFUS thalamotomy in thirty patients with tremor across a wide-range of disorders (18 with ET, 9 with PD and 3 with ET-PD) [13]. At six-month follow-up, patients with ET had a significant decrease of CRST score from 40.7 to 8.2; patient with PD or ET-PD had a significant decrease in motor component of PDQ-39 from 38.6 to 20.6. Quality of Life in Essential Tremor (QUEST) score similarly improved. Common adverse events following MRgFUS thalamotomy include paresthesia, taste disturbances, headaches, and reported sense of imbalance. While pin site discomfort from the stereotactic frame is common, no infection has been reported so far. More serious complications such as ataxia are approximately 1.6% [5]. Zaaroor et al. reported that all neurological complications improved by three months after treatment [13].

#### Parkinson’s disease

Parkinson’s disease (PD) is the second most common neurodegenerative disorder, with a prevalence of approximately 50 per 100,000 people at 65 years of age [14]. PD is characterized by motor symptoms such as tremor, bradykinesia, and rigidity, with tremor present in 50% of patients at the time of diagnosis [15]. Motor symptoms are caused by the progressive death of dopaminergic neurons in the substantia nigra pars compacta (SN) [16], and although a wide range of medications are used to manage PD, dopamine replacement is the mainstay of treatment. For patients who become refractory to medical treatment neurosurgical intervention may be appropriate, targeting critical motor nuclei that are pathophysiologically linked to the core motor symptoms of PD. Currently, the most widely practiced surgical treatment is DBS of subcortical motor structures, the globus pallidus internus (GPi) and subthalamic nucleus (STN) [10, 17]. While DBS is the gold-standard surgical approach to refractory PD that is still responsive to levodopa, the operation may not be suitable for every patient, nor every symptom. For example, older patients are often excluded from DBS, and the procedure may not be suitable for those who cannot undergo general anesthesia, or who suffer from levodopa induced dyskinesia. Accordingly, there is growing interest and experience in MRgFUS for PD, as an alternative to DBS and other open ablative procedures, such as RF pallidotomy [15]. Various targets have been proposed for MRgFUS ablation to treat tremor-dominant PD in early clinical studies, including the pallidothalamic tract [18], Vim thalamus, [13, 19, 20], and GPi [21]. The optimal target in PD is unclear, and will depend on the symptomology of the patient. Recent studies in PD patients treated with MRgFUS, report clinical significant improvement in motor symptoms after treatment ranging from 61% reduction in Unified PD Rating Scale (UPDRS) motor score at 3 months for pallidothalamic tractomy [18] to 46.2% at 6 months for Vim thalamotomy [13]. However, different methodologies and outcome measures make direct comparison difficult (For detailed discussion see Schlesinger et al. [15]). Future studies will need to compare outcomes after MRgFUS ablation compared to placebo or current surgical treatment options for tremor-dominant PD.

The potential of low intensity ultrasound in conjunction with microbubbles to safely and transiently open the blood brain barrier (BBB) has also attracted considerable interest by PD researchers. The BBB is the brain’s primary defense against large (> 400 Da) and hydrophilic molecules. Small molecules and certain drugs can pass through the BBB through diffusion or receptor mediated cellular transport [22]. However, the BBB has hampered therapeutic development for various neurological disorders such as PD and Alzheimer’s disease (AD) [23].

Multiple animal studies have shown that ultrasound mediated BBB disruption is temporary, reversible within hours, and safe to surrounding tissue at appropriately selected ultrasound parameters [24]. Interactions between ultrasound and pockets of gas or injected microbubbles lead to inertial cavitation, oscillations of the microbubbles, resulting in mechanical force on tight junctions and endothelial cells [25]. The extent of the BBB opening depends on the microbubbles (e.g. size, conjugates) as well as ultrasound parameters (e.g. intensity, sonication time) [26]. Preclinical studies ranging from mouse to nonhuman primates have demonstrated the safe delivery of macromolecules [27], viruses [28], and substances as large as cells [29, 30].

Randomized control trials of direct intraparenchymal inoculation of neurotrophic agents, such as glial cell-derived neurotrophic factor (GDNF), or embryonic dopamine neurons in degenerating dopaminergic pathways have largely failed to date [31–33]. One possible explanation could be inadequate delivery of therapeutic compounds across the blood brain barrier [34]. A growing preclinical MRgFUS literature has demonstrated increased intraparenchymal concentration of growth factors, stem cells, and gene therapy after BBB opening [30, 35–37]. In one study, done in a 6-OHDA model of PD, MRgFUS delivery of GDNF resulted in significantly higher levels of dopamine and less abnormal apomorphine-induced rotational behavior than after direct intraparenchymal infusion of GDNF [35].

### Pain

Neuropathic pain (NP) is complex, multifactorial, and difficult to treat. NP can originate from a dysfunction in the nervous system due to disease, injury, or iatrogenic causes. Prevalence of chronic pain with neuropathic features in the general population may be as high as 8% [38]. Primary management includes tricyclic antidepressants (TCAs), selective serotonin-norepinephrine reuptake inhibitors (SSRIs), anti-epileptic medications, and opioids, which may result in intolerable side effects and tolerance over time [39]. Various surgical strategies have been developed to treat chronic pain, including cordotomy, sympathectomy, myelotomy, dorsal root entry zone lesions, mesencephalotomy, and cingulotomy, depending largely on the nature of the pain, it’s location, and previous treatment attempts [40]. Ablative procedures in the thalamus have also been performed for several decades [41], and now MRgFUS offers a less invasive alternative. In one study, central lateral thalamotomy using MRgFUS in 12 patients suffering from chronic medically-refractory NP reported mean pain relief of 57% at 12 months [42]. Further clinical trials are underway to investigate the effects of MRgFUS thalamotomy for neuropathic pain (NCT01699477).

### Obsessive-compulsive disorder

Obsessive-compulsive disorder (OCD) is among the most challenging and debilitating anxiety disorders, with an estimated lifetime prevalence of 1.6% [43]. OCD is marked by unwanted intrusive, recurrent, and persistent thoughts, images, or urges that are often accompanied by time-consuming and repetitive behaviors. OCD has been linked to various neurochemical and neuroanatomical pathways, including hypersensitivity of post-synaptic serotonin receptors, and mutations in serotonin transporter and receptor genes [44–47]. Accordingly, first-line treatment has typically focused on brain chemistry, using selective serotonin reuptake inhibitors or tricyclic antidepressants such as clomipramine, paired with psychotherapeutic interventions, such as exposure and cognitive behavioral therapy [48]. However, despite optimal care, up to 30 to 40% of patients remain significantly disabled [49, 50]. Recent imaging, preclinical, neuropsychological, and treatment studies have increasingly supported the view that OCD is a disorder of neural circuitry [51]. For treatment resistant patients, therefore, surgical approaches which target specific brain regions and circuitry, may be an emerging option.

The anterior limb of the internal capsule (ALIC) has been closely associated with the surgical management of refractory OCD [52]. The ALIC contains a rich network of fibers that connect both the prefrontal cortex and anterior cingulate cortex with the hippocampus, amygdala, and thalamus [53–55]. Disruption of these tracts is believed to be important in treating refractory OCD, by disrupting limbic cortico-thalamic fibers affecting depression and anxiety and influencing circuits critical for emotion and affective processing [53–57]. A meta-analysis of DBS and open label prospective anterior capsulotomy studies in patients with refractory OCD found a 51% reduction on the Yale-Brown Obsessive Compulsive Scale (Y-BOCS) after ablation (radiofrequency and gamma-knife radiosurgery) compared to 40% after DBS [58]. Furthermore, individuals who underwent the ablative therapy were 9% more likely to go into remission than DBS. A recent systematic review of eight observational studies with a total of 112 patients after AC showed a mean reduction of 55% on the Y-BOCS at 12 months, superior to results from meta-analysis of outcomes after dorsal anterior cingulotomy [59].

Non-invasive approaches to capsulotomy include gamma knife radiosurgery (GKRS) and MRgFUS. GKRS uses ionizing radiation for incision-less ablation, but results have a latency period of weeks to months [60]. Additional concerns about exposure to ionizing radiation include risk of neoplasm and injuries to off target structures [61]. The effect of MRgFUS is immediate, permitting real-time monitoring of the lesion and the temperature required to achieve it. Jung et al. reported the proof-of-concept study of bilateral thermal capsulotomy using MRgFUS in four patients with medically refractory OCD [62]. Gradual improvements in Y-BOCS score were noted over 6 months, with an ultimate mean improvement of 33%. These patients also had comorbid major depressive disorder. Over the same period, patients experienced sustained reductions in symptoms of depression (mean 61.1%) and anxiety (mean 69.4%). No adverse effects or significant differences in neuropsychological test scores were noted. This result is promising, but durability of the outcome, cumulative safety profile, and its value over placebo or traditional nonablative methods (e.g. DBS) and ablative methods (e.g. GKRS) will require further investigations.

The development of MRgFUS ablation as a therapy for other psychiatric illnesses are also underway. The first MRgFUS procedure for treatment-resistant major depressive disorder (MDD) showed initial safety and improvement in the Hamilton Depression Rating Scale score from 26 pre-procedure to 7 at 12 months, Beck Depression Inventory score from 26 to 12 at 12 months [63]. The lesion target for treatment-resistant MDD is also ALIC. This single case report demonstrated marked clinical improvement. Two clinical trials (NCT02348411, NCT02685488) are currently underway to further define the role of ultrasound in depression.

### Brain tumors

The most common brain tumors, metastatic and glioblastoma multiforme (GBM), have prognoses of 3 [64] and 4 months survival, respectively, if left untreated [65]. Indeed, despite advances in genetics and concerted multidisciplinary efforts in developing new therapeutics, the gains in patient survival have been modest. With modern treatment, the median overall survival of patients with GBM is only 14.6 months [66]. Tumors in eloquent or deep brain regions are poor candidates for surgical resection and, though traditionally treated with radiotherapy, may benefit from alternative modalities now under investigation, including proton beam radiation and laser interstitial thermal therapy (LITT) [67]. MRgFUS may represent an additional option.

#### Ablation

Focused ultrasound thermoablation of bone metastases, prostate cancer, and uterine fibroids are approved by the FDA [34, 68, 69]. At temperatures above approximately 55 °C, tumor tissue shows homogenous coagulative necrosis leading to irreversible cell death [70]. Early clinical studies of MRgFUS thermoablation for brain tumors have predominantly focused on high-grade gliomas. McDannold et al. published the first experience with sonication of high-grade glioma through the intact skull [71]. While the procedure proceeded without any complications seen in previous studies (e.g. thermal injury in the pre-tumor area), coagulative necrosis was not achieved due to insufficient power. As such, the adjustments including doubling the number of transducer elements, decreasing the frequency from 650 kHz to 230 kHz and increasing the power capabilities were made to the system. Unfortunately, the fourth patient suffered a post procedure intracranial hemorrhage, which was linked to possible underlying coagulopathy and inertial cavitation. This complication highlighted the challenges of ablating a large volume and vascular neoplastic tissue as well as greater attention to patients or pathologies with increased risk of hemorrhage. Currently phase I trials (NCT01698437, NCT00147056, NCT01473485) are underway with results pending.

Targets located close to the skull are difficult to thermoablate without unacceptable rise in temperature in bone, scalp, or neighboring neurovascular structures. Recently, two proof of concept animal studies of non-thermal ablation of skull base lesions were reported. McDannold et al. demonstrated that low-duty cycle sonication at 525 kHz with intravenously injected microbubbles can produce lesions at the skull base close to the optical nerve in rats [72]. Sonicated microbubbles undergo inertial cavitation, leading to vascular injuries and ischemic necrosis in the target. Evidence from histology and evoked potential data supports preservation of the optic nerve. This study was replicated in nonhuman primates using MRgFUS operating at 220 kHz, low duty cycles and intravenous Definity® microbubble contrast [73]. However, off-target effects included unintentional BBB opening, particularly in the pre-tumor area. Further safety studies are necessary to examine the tradeoff to this approach, a greater risk of inertial cavitation along the acoustic path [74].

#### Blood brain barrier disruption for chemotherapy delivery

While tumor cells can break down the blood barrier, they also infiltrate into surrounding tissue resulting in angiogenesis and blood vessels with an intact BBB [75]. A majority of chemotherapies have limited bioavailability in the brain and tumor tissue. Additionally, while targeted therapies for specific cancers (e.g. trastuzumab for HER2 positive breast cancer and vemurafenib, BRAF kinase inhibitor, for melanoma) have dramatically changed the prognosis of patients without intracranial metastasis, these drugs poorly penetrate the BBB [76, 77]. As a result, several attempts have been made to couple chemotherapy with transient opening of the BBB. For example, simultaneous intra-arterial administration of mannitol and carboplatin or bevacizumab infusion has been examined in early clinical trials to treat high grade gliomas [78]. In addition to mixed and modest outcomes, patients were exposed to potential complications from the endovascular procedure and neurotoxicity from nonspecific brain uptake of the chemotherapy. MRgFUS offers a less invasive approach to open the BBB transiently with enhanced spatial and temporal specificity.

An early animal study demonstrated successful and safe delivery of trastuzumab in mouse [27]. In another study, ultrasound mediated BBB opening plus temozolomide (TMZ) administration compared to TMZ alone in rats with implanted 9 L glioma cells reduced 7-day tumor progression and extended median survival from 20 to 23 days [79]. CSF-to-plasma ratio of TMZ level was increased by 1.7 times. In another study, eight treatments of ultrasound delivered NK-95 cells in mice injected with human HER2-expressing breast tumor resulted in an increase in overall survival from approximately 50 to 150 days [29]. As the result of compelling preclinical data, a pilot trial is underway to investigate safety and feasibility of ultrasound mediated BBB disruption for patients with high-grade gliomas to deliver TMZ (NCT02343991).

### Alzheimer’s disease

Alzheimer’s disease (AD) is the most common neurodegenerative disease, affecting more than five million people in the United States, and increasing. The pathologic hallmarks of AD consist of beta-amyloid plaques and neurofibrillary tangles resulting in synaptic dysfunction and neurodegeneration. Current FDA approved medications, anti-cholinesterase inhibitors and NMDA receptor antagonist, have only modest clinical benefit. Alternatively, biologic agents, such as bapineuzumab and solanezumab, aim to improve clearance of Aβ plaques and oligomers [80]. While they have proven to be effective both histologically and behaviorally in animal models and early clinical trials, there has yet to be an immunotherapy proven to be clinically effective in phase 3 clinical trials [81, 82].

As with PD and Brain tumors, the BBB is a major obstacle to the effective delivery of potential therapeutic compounds to AD brains, and MRgFUS has been explored as a possible means to overcome it. In a transgenic mouse model of AD, treated with ultrasound delivered, anti-BAM, an antibody against Ab plaque, to a single hemisphere results in a reduction of plaque number and surface area by more than 20% compared to the contralateral hemisphere after four days [83]. After treatment, transgenic animals performed as well as wild-type animals on the Y maze task, suggesting a rescue of spatial memory deficits. Surprisingly, BBB opening alone also led to a significant plaque reduction. Increased endogenous IgG and IgM antibodies bound to beta amyloid plaques and glial activation after BBB opening suggests that the influx of endogenous antibodies can lead to activation of the innate immune system against the pathologic plaques. Moreover, an increased hippocampal neurogenesis was found after sonication, which may also contribute to the memory rescue.

Reductions in plaque number and surface area after ultrasound mediated BBB opening alone were replicated by another research group in APP23 transgenic mouse line [84], along with behavioral improvement as well as a five-fold reduction of Aβ monomer and two-fold reduction of Aβ trimer, which may have an even greater role in AD pathogenesis. More recently, the same group demonstrated ultrasound delivered RN2N, an antibody to tau, reduced phosphorylated tau levels in a transgenic TauP301L mouse [85]. The presence of neurofibrillary tangles and hyper-phosphorylated tau are closely correlated with the progression of cognitive deficits in patients with AD. An ongoing phase I trial will study the feasibility and safety of BBB opening in patients with mild Alzheimer’s disease (NCT02986932).

## Conclusion and future directions

MRI guided focused ultrasound can be used at high and low intensities for neuroablation, opening the BBB and potentially neuromodulation. We reviewed the clinical applications of neuroablation and BBB opening that are established or currently under investigation in human studies. Although promising, several challenges exist for brain applications of MRgFUS. For example, targets close to the skull are challenging to treat with high frequencies, given the bone heating that results from the large amount 0of energy necessary to achieve coagulative necrosis. Non-thermal ablation may circumvent this problem [72], although the experience is preliminary and can be associated with unintended off-target changes (e.g. BBB opening) [6].

Further, the relative efficacy of MRgFUS ablation for tremor, and other indications such as OCD, compared to traditional open surgical approaches will need to await longer follow-up and larger clinical experiences. Current treatment times require several hours in the MRI machine, and improvements in sonication protocol and workflow are needed to decrease treatment time and enhance tolerability. Similarly, technologic advances eliminating the need for complete head shave and the use of a stereotactic frame, will contribute to making the procedure more tolerable for patients.

Basic scientists will continue to study how ultrasound interacts with tissue. Questions exist as to the chronic changes to the neurovascular unit after BBB opening and how they will affect repeat treatments. A recent study reported that ultrasound mediated BBB opening leads to a sterile inflammatory reaction [86]. Although inflammation is expected after mechanical opening of the BBB and may in fact contribute to clearance of beta amyloid plaques, further studies are needed to quantify the cellular and molecular genetics changes from BBB opening at minimal energy settings. On the clinical front, researchers are exploring the potential of combining MRgFUS mediated BBB opening with drugs or biologic agents. In doing so, FUS could be a valuable tool in treating a wide range of neurological conditions, such as neurodegenerative diseases and demyelinating diseases, but phase I studies will need to first establish the safety and feasibility of BBB opening in these populations. As research teams are poised to translate existing animal data to human studies in the next decade, there remains promise that intracranial MRgFUS can have an increasingly important role in the management of the most challenging brain conditions.

## Abbreviations

AD: Alzheimer’s disease
BBB: blood brain barrier
CRST: clinical rating scale for tremor
DBS: deep brain stimulation
ET: essential tremor
FUS: focused ultrasound
GDNF: glial cell line derived neurotrophic factor
GKRS: gamma knife radiosurgery
GPi: globus pallidus interna
HER2: human epidermal growth factor receptor 2
LITT: laser interstitial thermal therapy
MDD: major depressive disorder
MRgFUS: magnetic resonance image guided focused ultrasound
NP: neuropathic pain
OCD: obsessive compulsive disorder
PD: Parkinson’s disease
QUEST: quality of life in essential tremor
RF: radiofrequency
SN: substantia nigra
TMZ: temozolomide
UPDRS: unified Parkinson’s disease rating scale
Vim: ventral immediate nucleus of the thalamus
Y-BOCS: Yale-Brown Obsessive Compulsive Scale
